# How to Be Happy

**Published:** 1855-10

**Authors:** 


### HOW TO BE HAPPY.

I sometimes think that I give too much good advice in my Journal for a paltry dollar; however, as there is plenty more where that came from, and *there is that scattereth and yet increaseth*, I will continue to distribute liberally, especially as I feel so full of it sometimes, that I am almost tempted to double the size of this little pet of mine. Yet "*second thoughts*" suggest that I had better explode myself than to explode the Bank, for then all three of us, Journal, Bank, and I, would grave together.

*But how to be happy?* that is the question. Reader, I have seen a great deal and felt more; have talked, and travelled, and enjoyed, and suffered, with all sorts of people; have wandered much and staid at home more; have been on the sea, and in it, and under it; have been laughed at, shot at, quarrelled at, praised, blamed, abused; have been blown at, and blown up; have had much, and had little, so much as to enjoy nothing; so little that I would have enjoyed a crust of bread, because the ship went to the bottom with everything in it, leaving me to float to a sand bank; and then again I have wandered over the earth, and under it, and through it, its caves, and its dungeons and darkness, after stalagmites and stalagtites, and specimens of black rocks and white ones, blue stones and gray; lived for months on desert islands, just for the purpose of picking up new shells on the beach, which the tide of night never failed to leave behind it; in those bygone days, when I had the three great requisites of an enjoying traveller, to wit: plenty of time, plenty of patience, and plenty of money, so if the coach turned over and smashed up, I could afford to wait until another could be had, or if the ship went to the bottom instead of to its destined port, 'twas just the same to me, because if I wasn't at one place I was at another, and there was always some strange rock to look at, some queer "*dip*," that set me calculating how many horse power it required to make that rock *just turn up so*, and all the million inquiries which geology, astronomy, conchology, and a dozen other dry names suggested, which not only had the effect to keep me from fretting, but kept me in an interested humor; well, in all these different situations, and as many more, I have found out, among others, three things:

1st. That a man out of money can't be happy.

2d. That a man out of health can't be happy.

3d. That a man without a wife can't be happy.

Therefore, I have come to the conclusion, that *the best way to be happy is to take care of your health, keep out of debt, and get a wife.*

